# Quantum dots-based double imaging combined with organic dye imaging to establish an automatic computerized method for cancer Ki67 measurement

**DOI:** 10.1038/srep20564

**Published:** 2016-02-03

**Authors:** Lin-Wei Wang, Ai-Ping Qu, Wen-Lou Liu, Jia-Mei Chen, Jing-Ping Yuan, Han Wu, Yan Li, Juan Liu

**Affiliations:** 1 Department of Surgical Oncology, Beijing Shijitan Hospital Affiliated to the Capital Medical University, Beijing, 100038, China; 2 Department of Oncology, Zhongnan Hospital of Wuhan University, Wuhan 430071, China; 3 School of Computer Science and Technology, University of South China, Hengyang, 421001, China; 4 Department of Pathology, Renmin Hospital of Wuhan University, Wuhan 430060, China; 5 School of Computer, Wuhan University, Wuhan 430072, China

## Abstract

As a widely used proliferative marker, Ki67 has important impacts on cancer prognosis, especially for breast cancer (BC). However, variations in analytical practice make it difficult for pathologists to manually measure Ki67 index. This study is to establish quantum dots (QDs)-based double imaging of nuclear Ki67 as red signal by QDs-655, cytoplasmic cytokeratin (CK) as yellow signal by QDs-585, and organic dye imaging of cell nucleus as blue signal by 4′,6-diamidino-2-phenylindole (DAPI), and to develop a computer-aided automatic method for Ki67 index measurement. The newly developed automatic computerized Ki67 measurement could efficiently recognize and count Ki67-positive cancer cell nuclei with red signals and cancer cell nuclei with blue signals within cancer cell cytoplasmic with yellow signals. Comparisons of computerized Ki67 index, visual Ki67 index, and marked Ki67 index for 30 patients of 90 images with Ki67 ≤ 10% (low grade), 10% < Ki67 < 50% (moderate grade), and Ki67 ≥ 50% (high grade) showed computerized Ki67 counting is better than visual Ki67 counting, especially for Ki67 low and moderate grades. Based on QDs-based double imaging and organic dye imaging on BC tissues, this study successfully developed an automatic computerized Ki67 counting method to measure Ki67 index.

Cancer is the leading cause of death and a heavy burden to the global health^1^,^2^. Although there have been great progresses in screening tools and comprehensive therapies, cancer mobility and mortality is still rising, especially in developing countries^1^,^3^,^4^. Currently, recurrence and metastasis are major causes of cancer death, which are mainly due to high cancer cells proliferation and migration^5^. Therefore, great efforts have been made to explore cancer proliferation markers for predicting cancer prognosis and formulating more individualized treatments. Many proliferative markers, such as proliferating cell nuclear antigen, Ki67, cyclin D, cyclin E, p27 and p21 have been discovered^6^,^7^,^8^. Among these proliferative molecules, Ki67 is a widely used marker in clinical cancer treatment and prognosis^9^,^10^,^11^,^12^,^13^, and can specifically label proliferative cells at mitotic phase^14^. And it has been recommended as a key marker of breast cancer (BC) subtypes by the St Gallen International Expert Consensus in 2011^15^ and gastroenteropancreatic neuroendocrine tumors grading system by World Health Organization^16^.

Accurate counting of Ki67 is the key to appreciating its prognostic value. However, Ki67 counting has always been a formidable challenge for cancer research due to variability of Ki67 expression and complexity of tumor tissue^6^,^10^,^17^. Currently, the frequently used measurement of Ki67 index is based on immunohistochemical (IHC) staining tissue sections, which can be briefly divided into two mainstream methods: artificial method and computerized method^10^. The former is the common method of manual counting and visual estimation. Manual counting requires precisely measure Ki67 positive cells in as many as 1,000 cancer cells from at least 3 high-power fields at the invasive edge, which is the most frequently used method in clinical practice as recommended by the International Ki67 in Breast Cancer Working Group^9^. The visual estimation, also known as “eyeballing”, is to rapidly count a global subjective Ki67 index by quick scanning and rough estimating the global cancer section^18^,^19^,^20^. However, the two manual methods are highly experience-based and subjective, which are criticized due to their intra- and inter-observer variations for the same tissue section even among the experienced pathologists^10^,^17^.

The recently developed automatic Ki67 counting is considered as a promising alternative method for Ki67 scoring. Different from traditional computer software, which only obtained Ki67 sum of the specific tumor tissue (the Ki67 sum is influenced by the number of cancer cells in the analytic targets)^21^, the new automatic Ki67 counting methods aimed at recognizing/counting both Ki67-positive and negative cancer cells, thus making the Ki67 counting more objective, efficient, and accurate^12^,^18^,^22^,^23^,^24^. The computerized methods show properties of objectivity, speediness and high repeatability, and have stronger prognostic value than manual counting^18^,^20^,^22^,^25^. However, the accuracy of these methods is also questioned because the computerized recognition of Ki67 positive and negative cancer cell nuclei was only based on the features of cell nuclei such as color, shape and size, and it is difficult to eliminate interferences of stroma non-cancer cells (such as lymphocyte cells, fibroblast cells, macrophage cells)^10^. Therefore, measurement of Ki67 index in cancer remains a formidable challenge for both oncologists and pathologists^6^,^10^,^17^.

The key to computer-aided automatic Ki67 measurement is to recognize Ki67 positive and negative cancer cells in complex background of heterogeneous tumor tissue images. Preferably, information on Ki67-postive cells, cancer cells, and stroma cells should be specifically labeled and accurately recognized and counted. A pixel-based clustering algorithm has been developed by Gustavson *et al*.^26^ to automatically and quantitatively analyze targets stained with immunofluorescence, which was applied to automatically assess Ki67 index in oral cancer^13^,^27^. However, multiple immunofluorescent staining could not avoid interference of tissue autofluorescence and mutual interferences of multiple organic dyes. Moreover, how to simultaneously obtain such multi-dimensional information from complex tumor tissue is also a technique challenge for organic immunofluorescent imaging technique^28^.

QDs are semiconductor nanocrystals with excellent optical properties, such as high fluorescence intensity, strong resistance to photobleaching and chemical degradation, size-tunable emission wavelength, and simultaneous multiple imaging under a single excitation source^28^,^29^,^30^. Because of these optical advantages, QDs-based imaging has been widely applied in cancer researches to study biomarkers interactions, evaluate prognostic biomarkers, map axillary lymphatic system, show xenograft tumor, and detect cancer metastases^28^. We have established QDs-based multiple imaging in tumor tissue sections^31^ and demonstrated QDs-based imaging on Ki67 of BC showed good correlation and consistency with conventional IHC, with better image quality and sensitivity^32^. Additionally, QDs-based double imaging on Ki67 and cytokeratin (CK, an epithelial specific marker) of BC was successfully developed to show a superior indicator of Ki67/CK than Ki67 sum to predict BC prognosis^10^.

This study is aimed at developing an automatic computerized Ki67 index recognition and measurement method by integrating QDs-based multiple imaging of Ki67 and CK with organic dye imaging of 4′,6-diamidino-2-phenylindole (DAPI) in BC.

## Results

### Typical images of different Ki67 indexes counted by computerized method

**Figure 1:**
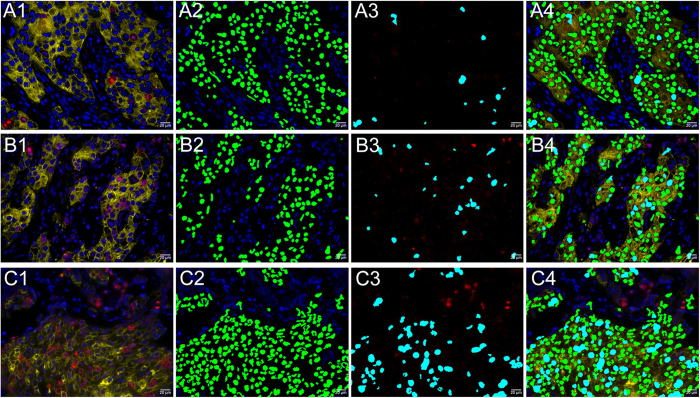
Typical examples of different Ki67 indexes counted by computerized method. Original images, recognized images of cancer cells, recognized images of Ki67-positive cancer cells, and merged images of recognized cancer and Ki67-positive cancer cells for BC with Ki67 low grade (**A1–A4**), moderate grade (**B1–B4**), and high grade (**C1–C4**). (Magnifications: 400×).

To clearly show the results of computerized method, we selected three typical images with Ki67 low, moderate and high grades counted by computerized method (Fig. 1). Figure 1A1–C1 were original BC images of Ki67 low, moderate, and high grades. These images could clearly showed red Ki67 positive cell nuclei, yellow cytoplasm and blue cell nuclei. Figure 1A2–C2 were computer-recognized BC images of cancer cells with Ki67 low, moderate, and high grades. These images showed cancer cell nuclei were recognized by the computer with green pseudocolor, but stromal cell nuclei were not. Figure 1A3 were computer-recognized BC images of Ki67-positive cancer cells with Ki67 low, moderate, and high grades. These images showed Ki67-postive cancer cell nuclei were recognized by the computer with cyan pseudocolor, but Ki67-positive stromal cell nuclei were not. Figure 1A4showed merged images of recognized cancer cells and Ki67-positive cancer cells with Ki67 low, moderate, and high grades. These images showed cell nuclei of red Ki67 staining and blue DAPI staining outside of yellow CK staining were not recognized by the computerized method. These results indicate the computerized method could well recognize Ki67 index of different grades.

### Results of marked Ki67 (M-Ki67) counting, computerized Ki67 (C-Ki67) counting and visual Ki67 (V-Ki67) counting

In the background of CK stained area to outline the cancer region, BC cells with DAPI staining and Ki67-positive cells with QDs-655 staining are clearly delineated, and were counted by marked method, computerized method and visual method, respectively. The Ki67 counting results by these three methods, divided by Ki67 grading, were listed in Table 1.

### Comparisons of M-Ki67, C-Ki67 and V-Ki67 indexes by analysis of variance (ANOVA)

ANOVA was conducted to compare Ki67 indexes by M-Ki67, C-Ki67 and V-Ki67 counting (Table 2). There was no statistical difference between M-Ki67 and C-Ki67 for low (*P* = 0.725), moderate (*P* = 0.236), and overall grades (*P* = 0.288). Likewise, there was no statistical difference between M-Ki67 and V-Ki67 for Ki67 low (*P* = 0.060) and overall grades (*P* = 0.068). And, there was no statistical difference between M-Ki67 and V-Ki67 for Ki67 moderate (*P* = 0.513) and overall grades (*P* = 0.082). Moreover the results in Table 2 indicate the differences between M-Ki67 and C-Ki67 were lower than those between M-Ki67 and V-Ki67 for each Ki67 grade and overall grades.

### Consistency analysis of M-Ki67, C-Ki67 and V-Ki67 indexes by intraclass correlation coefficient (ICC) test

ICC test was performed to explore inter-observer consistency of M-Ki67, C-Ki67 and V-Ki67 indexes (Table 3). For overall grades, M-Ki67 *vs.* C-Ki67 (ICC = 0.964, *P* < 0.001), M-Ki67 *vs.* V-Ki67 (ICC = 0.923, *P* < 0.001), and C-Ki67 *vs.* V-Ki67 (ICC = 0.919, *P* < 0.001) had good consistency. The consistencies of M-Ki67 *vs.* C-Ki67 in low (ICC = 0.662, *P* = 0.002), moderate (ICC = 0.755, *P* < 0.001) and high grades (ICC = 0.575, *P* = 0.012) were higher than those of M-Ki67 *vs.* V-Ki67 in low (ICC = 0.585, *P* = 0.010), moderate (ICC = 0.570, *P* = 0.013) and high grades (ICC = 0.494, *P* = 0.036). Only the consistency of C-Ki67 *vs.* V-Ki67 in low grade (ICC = 0.320, *P* = 0.153) has no statistical significance. The results indicate that C-Ki67 has higher consistency than V-Ki67 in each Ki67 grade.

### Spearman correlation analysis of M-Ki67, C-Ki67 and V-Ki67 counting

The correlations among M-Ki67, C-Ki67 and V-Ki67 counting were tested by Spearman analysis (Table 4). The correlation coefficient of M-Ki67 *vs.* C-Ki67 for Ki67 low (r = 0.671, *P* < 0.001), moderate (r = 0.628, *P* < 0.001) and overall grades (r = 0.932, *P* < 0.001) was higher than those of M-Ki67 *vs.* V-Ki67 for Ki67 low (r = 0.413, *P* = 0.023), moderate (r = 0.415, *P* = 0.023) and overall grades (r = 0.895, *P* < 0.001). For Ki67 high grade, the correlation coefficient of M-Ki67 *vs.* C-Ki67 (r = 0.370, *P = *0.044) was lower than that of M-Ki67 *vs.* V-Ki67 (r = 0.378, *P* = 0.039). And correlation coefficients of C-Ki67 *vs.* V-Ki67 for Ki67 moderate (r = 0.522, *P* = 0.003) and overall grades (r = 0.874, *P* < 0.001) had statistical significance. Those results reveal that in specimens with either low or moderate Ki67 grades, C-Ki67 counting results will have greater consistency than V-Ki67 counting.

## Discussion

**Figure 2:**
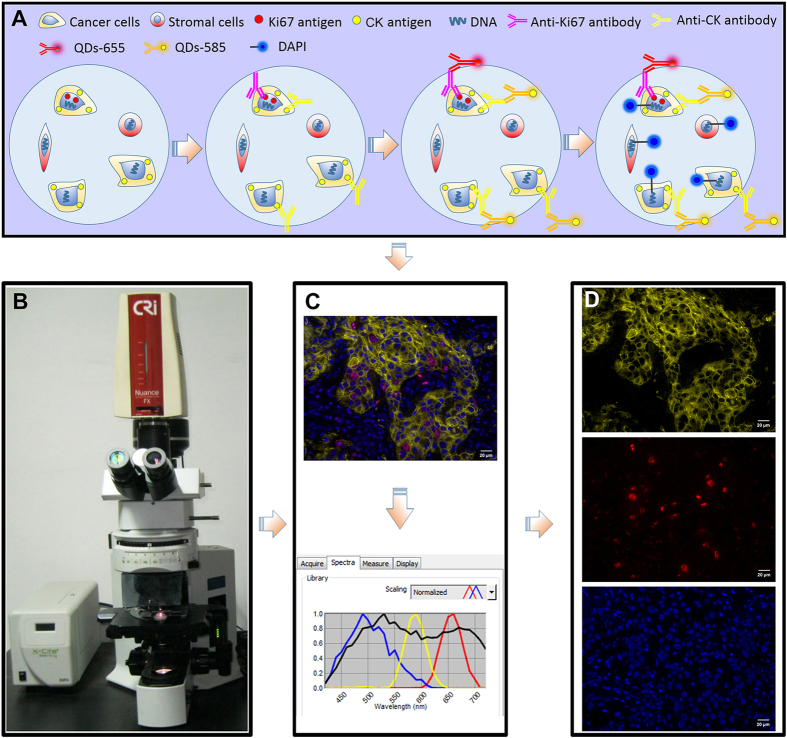
Major procedures of multiplexed imaging and image acquisition for this study. QDs-based double imaging and DAPI imaging for BC tissues (**A**), information acquisition of multiplexed imaging (**B,C**) and information unmixing of multiplexed imaging (**C,D**).

In this study, we have developed a three-color simultaneous imaging of cancer cell proliferation marker Ki67, cancer cell cytoplasm CK and all cell nuclei (Fig. 2A). CRi Nuance multispectral imaging system was applied to simultaneously obtain an integrated image of 3 markers of interest in one imaging procedure, rather than the merged images of 3 individual colors (Fig. 2B,C). After eliminating non-target tissue autofluorescence, the integrated image was unmixed into three separate images of CK with yellow signal, Ki67 with red signal and DAPI with blue signal (Fig. 2C,D). Compared with fluorescent multiple imaging developed by Klimowicz *et al*.^13^,^27^, this method has two improvements. First is the improvement in imaging technique. QDs are semiconductor nanocrystals with excellent optical properties of narrow emission and wide excitation spectrum, a property favoring multiplexed imaging under one excitation spectrum without mutual interference of targeted signals of CK and Ki67^28^,^29^,^30^. This technical improvement could help accurately locate the markers of interest^33^. Second is the improvement in image information acquisition and analysis. The CRi Nuance multispectral imaging system guarantees that the information simultaneously obtained is the spectral signals of Ki67, CK and DAPI, further eliminating the interference of tissue autofluorescence and noisy signals with similar color but different spectrum^10^. These two improvements made our work a step forward to improving the precision and automatic process of Ki67 measurement.

**Figure 3:**
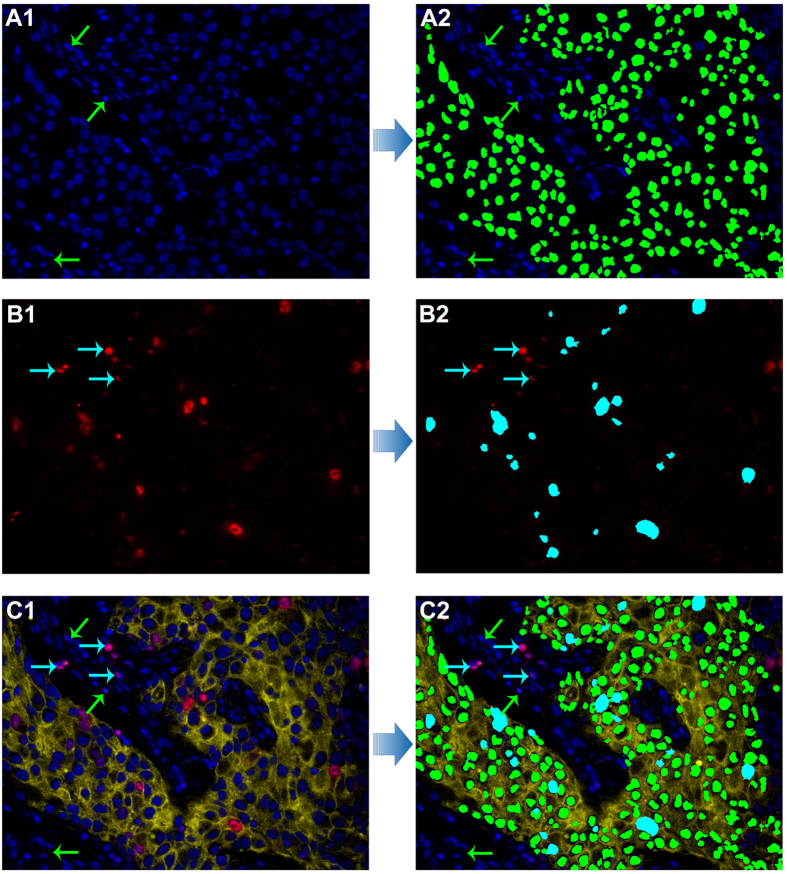
The technical procedures of Ki67 counting by computerized method. Recognition and counting of cancer cells with DAPI staining in CK stained area, as green pseudocolor (**A1,A2**) and cancer cells with Ki67 staining in CK stained area as cyan pseudocolor (**B1,B2**) both by the developed computerized method; the original image of CK, Ki67 and DAPI imaging for BC (**C1**) and merged image of recognition and counting of cancer cells with DAPI staining and Ki67 staining (**C2**). Stromal cells marked with green arrows (**A1,A2, C1** and **C2**) and stromal Ki67-positive cells marked with cyan arrows (**B1, B2, C1** and **C2**) were not recognized by the computerized method. (Magnifications: 400×).

Based on our previously developed computerized pathological image analysis approaches^33^,^34^,^35^, we developed a computerized method to automatically recognize and count Ki67 index in this study. The computer-recognized processes were briefly demonstrated in Fig. 3. These images showed cancer cell nuclei were recognized by the computer with green pseudo-color, but stromal cell nuclei were not (Fig. 3A1,A2), and Ki67-postive cancer cell nuclei were recognized by the computer with cyan pseudo-color, but Ki67-positive stromal cell nuclei were not (Fig. 3B1,B2). The merged images of recognized cancer cells and Ki67-positive cancer cells showed cell nuclei with red Ki67 staining and blue DAPI staining outside the yellow CK staining background were not recognized by the computerized method (Fig. 3C1,C2). Compared with computerized methods developed based on IHC staining^12^,^18^,^23^,^25^,^36^, the multiple imaging method could specifically label cell nuclei, cancer cell and Ki67 positive cell nuclei, which can assist computer to define and recognize cancer cell nuclei and Ki67 positive cancer cell nuclei, and eliminate the interference of stroma cells. Any blue signal outside the yellow background of CK is not cancer cell, as indicated by the green arrow in Fig. 3C1. Similarly, any red signal outside the yellow background of CK is not cancer cell Ki67, as indicated by the cyan arrow in Fig. 3C1. Therefore, this technical improvement could help accurately locate the markers of interest.

**Figure 4:**
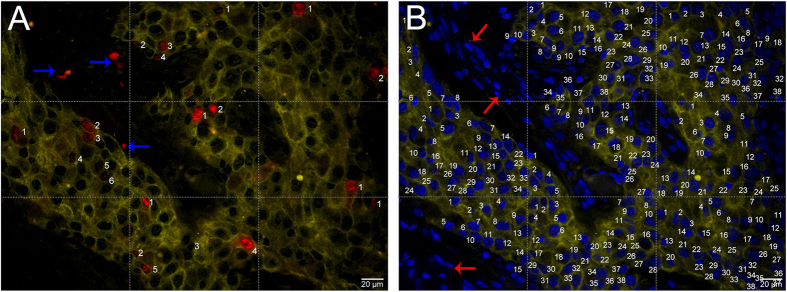
BC Ki67 counting by marked method. The merged images of Ki67 and CK (**A**), DAPI and CK (**B**) were divided into 9 equal parts; the numbers of Ki67 and DAPI only in CK-positive area for each part were counted by marked method, but Ki67 (blue arrows, **A**) and DAPI (red arrows, **B**) not in CK-positive area were ignored; a very accurate M-Ki67 index (total number of Ki67 in CK-positive ÷ total number of DAPI in CK-positive) was produced. (Magnifications: 400×).

To validate the performance of the C-Ki67 counting, we performed the comparisons of C-Ki67, M-Ki67 and V-Ki67 counting methods. In this study, M-Ki67 counting was a very carefully counting method to count Ki67 index as accurately as possible (Fig. 4), and was taken as the golden standard of Ki67 index. Results of M-Ki67, C-Ki67 and V-Ki67 counting for BC images were showed in Table 1. ANOVA results did not reveal statistical difference in M-Ki67 *vs.* C-Ki67 (*P* = 0.288) for overall grades, but there was statistical significant differences in M-Ki67 *vs.* V-Ki67 (*P* = 0.005) for overall grades (Table 2). The ICC test indicated consistencies in M-Ki67 *vs.* C-Ki67 were higher than those in M-Ki67 *vs.* V-Ki67 for all grades (Table 3). Spearman analysis indicated that the correlation coefficient was also higher in M-Ki67 *vs.* C-Ki67 than M-Ki67 *vs.* V-Ki67 for all grades besides high grade (Table 4). Similar to computerized methods established based on IHC staining^18^,^36^, the above results from this study demonstrated that C-Ki67 counting is better than V-Ki67 counting. At least, the counting performances of computerized methods are comparable to visual or manual counting methods^12^,^23^,^25^. Therefore, C-Ki67 could be considered as a sound substitution to M-Ki67. This could help increase the evaluation efficiency while maintaining the evaluation accuracy. The results also indicate that the current V-Ki67 counting method is not accurate, and it has increased efficiency but at the cost of reduced accuracy.

Although the integrated strategy of imaging and computerized method developed in this study showed advantages in automation, efficiency, consistency and better counting performance, a limitation should also be mentioned. Since this work at the current stage is focused on technical development, the prognostic significance of Ki67 index counted by the integrated strategy is not fully evaluated. In the future, more BC samples with completely clinic-pathological and prognostic information should be studied to verify the significance of C-Ki67 in prognosis and treatment of cancer.

## Conclusion

Based on the techniques of QDs double imaging and organic dye imaging, this study successfully developed a computerized image recognition and analysis system to automatically count Ki67 index of BC. Compared with visual counting method, this integrated strategy could improve the accuracy and efficiency of Ki67 index evaluation in cancer.

### Patients and Methods

#### Specimens

The fresh breast invasive ductal carcinoma formalin-fixed paraffin-embedded specimens tissue sections (thickness: 4 μm) from 30 patients with different Ki67 index (10 patients with low grade of Ki67 index ≤ 10%, 10 patients with moderate grade of Ki67 index > 10% and < 50%, and 10 patients with high grade of Ki67 index ≥ 50%) independently interpreted by two BC pathologists with rich experience based on IHC Ki67 staining were selected for this study. The technical procedures of all BC tissue specimens were performed by routine standardized pathological methodology in validated clinical laboratory to guarantee the consistency of pre-analytical issues. The study was approved by the Institutional Ethics Committee of Zhongnan Hospital of Wuhan University and undertaken according to the ethical standards of the World Medical Association Declaration of Helsinki. The written informed consent was obtained from the patients prior to operation to use tissue samples for scientific researches.

#### Multiplexed imaging of CK, Ki67 and DAPI

Multiplexed imaging of CK, Ki67 and DAPI was divided into two parts (Fig. 2A): QDs-based double imaging on CK and Ki67, and fluorescent imaging of DAPI. The major staining procedures were as following: BC sections heating → de-paraffinizing → hydration → antigen retrieval → blocking → primary antibodies for Ki67 and CK → staining with QDs-585 and QDs-655 simultaneously → washing → DAPI counterstaining. QDs-based double imaging has been well established at our center with detailed procedures in our previously published studies^10^,^31^,^37^. At first, the mixture of primary antibodies of anti-Ki67 rabbit monoclonal antibody (Clone: SP6; dilution: 1:100, Wuhan Jiayuan Quantum Dots Co., Ltd, China) and anti-CK mouse monoclonal antibody (Clone: AE1/AE3; dilution: 1:100, Wuhan Jiayuan Quantum Dots Co., Ltd, China) were applied to simultaneously recognize Ki67 and CK antigens in BC tissue sections at 4 °C overnight. Then, QDs-585 conjugated goat anti-mouse IgG (dilution: 1:200, Life Technologies, USA) and QDs-655 conjugated mouse anti-rabbit IgG (dilution: 1:600, Life Technologies, USA) was used to stain the yellow fluorescence of CK at the cytoplasm and the red fluorescence of Ki67 at the nucleus at 37 °C for 2 h. After QDs-based imaging, DAPI working solution was employed to counterstain cell nucleus for 10 min in the dark condition.

#### Image acquisition and unmixing

Fluorescence information on Ki67, CK and DAPI was simultaneously and randomly obtained based on 3 fields for each BC tissue sections under Olympus BX51 fluorescent microscope (Olympus Optical Co., Ltd. Tokyo, Japan) equipped with CRi Nuance multispectral imaging system (Cambridge Research and Instrumentation, Inc., Woburn, MA, USA) at 400 × magnifications. After imaging acquisition, the unmixing of information on Ki67, CK and DAPI was performed by the software package within CRi Nuance multispectral imaging system, which was divided into 2 major technical steps: (1) Selection of targets with different spectra: Ki67, CK, DAPI and tissue auto-fluorescence were selected as red, yellow, blue and black signals (automatically set by the software), respectively (Fig. 2B,C); (2) Image unmixing and elimination of background noise: the targets information with different spectra was automatically unmixed by the software based on specific spectrum into 3 separate images, i.e. CK image with yellow signal (Fig. 2D, upper panel), Ki67 image with red signal (Fig. 2D, middle panel), and DAPI image with blue signal (Fig. 2D, lower panel).

#### Ki67 counting by marked method

To count Ki67 index of BC tissues as accurately as possible, the merged images of Ki67 and CK, DAPI and CK were used to establish a M-Ki67 counting. After image acquisition and unmixing, merged images of Ki67 and CK (Fig. 4A), DAPI and CK (Fig. 4B) were divided into 9 equal parts by 4 dotted lines. Then, the numbers of Ki67 and DAPI only in CK-positive area with yellow QDs signals for each part were counted carefully by an expert pathologist with marked method, and verified by another pathologist. For each merged image part, only Ki67 and DAPI at the upper and left lines were counted, and those at the right and bottom lines of each part were ignored. This method produced a very accurate M-Ki67 counting (total number of Ki67 in CK-positive ÷ total number of DAPI in CK-positive) as the golden standard of Ki67 index in this study.

#### Ki67 counting by computerized method

A computerized method was developed to automatically count Ki67-positive cancer cell nuclei with red QDs signal and cancer cell nuclei with blue DAPI signal within cancer cell cytoplasm with yellow QDs signal (Fig. 3). First, pre-processing operations such as Median-Filter and contrast stretching were performed to denoise and adjust the contrast for improving image quality and signal unmixing. Second, the unmixed images were converted to gray-scales. The Ostu adaptive threshold method was used to obtain the binarization results. After morphological operations and flood-fill operation, the masks of blue DAPI of cell nuclei, red QDs of Ki67 and yellow QDs of CK were obtained, respectively. Since the obtained masks of the blue nuclei and red Ki67 positive nuclei contain connected clusters of nuclei, the marker-controlled watershed method was used to segment these two masks for identifying individual nucleus and post-processing procedures were used to correct the possible segmentation errors such as over or under-segmentation. Finally, blue cell nuclei and red Ki67-positive cell nuclei surrounded with yellow cell cytoplasm of CK staining were counted. The C-Ki67 index was the ratio of Ki67-positive cell nuclei and blue cell nuclei within yellow CK signal, which expressed as percentage. The recognition and counting of cancer cells with DAPI staining (Fig. 3A1), cancer cells with Ki67 staining in CK stained area (Fig. 3B,B2), and original image of CK, Ki67 and DAPI imaging (Fig. 3C1) and merged image of recognition and counting of cancer cells with DAPI staining and Ki67 staining (Fig. 3C2) were depicted in Fig. 3.

#### Ki67 counting by visual method

A subjective interpretation for the Ki67 index on the merged images of Ki67/CK and DAPI/CK was performed by two pathologists independently to provide a quantitative Ki67 index value of each image. The subjective visual method results of two pathologists for Ki67 index were defined as V-Ki67 with mean values.

### Statistical analysis

Statistical analysis was performed by SPSS 17.0 software (SPSS Inc. Chicago, IL, USA). M-Ki67, V-Ki67 and C-Ki67 indexes were expressed as percentages for each, and the differences of among the three results were tested by ANOVA analysis. The ICC consistency test and spearman correlation were used to analyze the consistency and correlation of M-Ki67, V-Ki67 and C-Ki67 counting. Two sided *P* < 0.05 was considered as statistically significant.

## Additional Information

**How to cite this article**: Wang, L.-W. *et al*. Quantum dots-based double imaging combined with organic dye imaging to establish an automatic computerized method for cancer Ki67 measurement. *Sci. Rep.* **6**, 20564; doi: 10.1038/srep20564 (2016).

## Figures and Tables

**Table 1:** Results of M-Ki67, C-Ki67 and V-Ki67 counting for 90 multiplexed images of 30 BC patients.

Ki67 grades (%)	Median M-Ki67 (range)	Median C-Ki67 (range)	Median V-Ki67 (range)
Low (≤10)	8 (1–12)	6.5 (1–21)	5.5 (2–13)
Moderate (>10 and <50)	26 (18–41)	23.5 (6–42)	21 (8–43)
High (≥50)	55.5 (45–74)	49 (30–71)	43 (25–68)
Overall grades	26 (1–74)	23.5 (1–71)	21 (2–68)

Results of Ki67 counting not located in defined ranges of Ki67 grades may be caused by following reasons: (1) There are variations in the Ki67 indexes of randomly acquired images; (2) There are variations in the Ki67 indexes of Ki67 counting methods.

M-Ki67: marked Ki67; C-Ki67: computerized Ki67; V-Ki67: visual Ki67.

**Table 2:** Comparisons of M-Ki67, C-Ki67 and V-Ki67 counting by ANOVA analysis.

Ki67 grades (%)	M-Ki67 *vs.* C-Ki67	M-Ki67 *vs.* V-Ki67	C-Ki67 *vs.* V-Ki67
Low (≤10)	*P* = 0.725	*P* = 0.060	*P* = 0.026
Moderate (>10 and <50)	*P* = 0.236	*P* = 0.068	*P* = 0.513
High (≥50)	*P* = 0.025	*P* < 0.001	*P = *0.011
Overall grades	*P* = 0.288	*P* = 0.005	*P* = 0.082

ANOVA: analysis of variance; M-Ki67: marked Ki67; C-Ki67: computerized Ki67; V-Ki67: visual Ki67.

**Table 3:** Consistency of Ki67 grade between C-Ki67index and M-Ki67 index by ICC test.

Ki67 grades (%)	M-Ki67 *vs.* C-Ki67	M-Ki67 *vs.* V-Ki67	C-Ki67 *vs.* V-Ki67
Low (≤10)	ICC = 0.662, *P* = 0.002	ICC = 0.585, *P* = 0.010	ICC = 0.320, *P* = 0.153
Moderate (>10 and <50)	ICC = 0.755, *P* < 0.001	ICC = 0.570, *P* = 0.013	ICC = 0.656, *P* = 0.003
High (≥50)	ICC = 0.575, *P* = 0.012	ICC = 0.494, *P* = 0.036	ICC = 0.488, *P* = 0.038
Overall grades	ICC = 0.964, *P* < 0.001	ICC = 0.923, *P* < 0.001	ICC = 0.919, *P* < 0.001

ICC: intraclass correlation coefficient; M-Ki67: marked Ki67; C-Ki67: computerized Ki67; V-Ki67: visual Ki67.

**Table 4:** Correlation analysis of M-Ki67,C-Ki67 and V-Ki67 counting by Spearman analysis.

Ki67 grades (%)	M-Ki67 *vs.* C-Ki67	M-Ki67 *vs.* V-Ki67	C-Ki67 *vs.* V-Ki67
Low (≤10)	r = 0.671, *P* < 0.001	r = 0.413, *P* = 0.023	r = 0.182, *P* = 0.337
Moderate (>10 and <50)	r = 0.628, *P* < 0.001	r = 0.415, *P* = 0.023	r = 0.522, *P* = 0.003
High (≥50)	r = 0.370, *P* *=* 0.044	r = 0.378, *P* = 0.039	r = 0.324, *P = *0.080
Overall grades	r = 0.932, *P* < 0.001	r = 0.895, *P* < 0.001	r = 0.874, *P* < 0.001

M-Ki67: marked Ki67; C-Ki67: computerized Ki67; V-Ki67: visual Ki67.
